# Supplementary material for: The Aetiology of Tourette Syndrome and Chronic Tic Disorder in Children and Adolescents: A Comprehensive Systematic Review of Case-Control Studies
Source: Brain Sci. 2022 Sep 6;12(9):1202. doi: 10.3390/brainsci12091202 (PMC9496979; doi:10.3390/brainsci12091202)
Supplement: Supplementary file 1 [file brainsci-12-01202-s001.zip › brainsci-1854310-supplementary.pdf]

Table S1. Quality assessment of the included studies

Author (Year)	NOS values			Study quality
	Selection	Comparability	Exposure	
[26]	★★★☆	★☆	★★☆	Fair
[27]	★★★☆	☆☆	★☆☆	Fair
[42]	★★★☆	★☆	★★☆	Fair
[28]	★★★☆	★☆	★★☆	Fair
[43]	★★★☆	★☆	★★☆	Fair
[13]	★☆☆☆	★☆	★★☆	Fair
[34]	★★★★	★☆	★★☆	High
[14]	★★☆☆	★★	★★★	High
[15]	★★★★	★★	★★☆	High
[16]	★★★☆	★★	★★☆	High
[39]	★★★☆	★☆	★★☆	Fair
[41]	★★★☆	★☆	★★☆	Fair
[29]	★★★☆	★☆	★★☆	Fair
[17]	★★☆☆	★★	★★☆	Fair
[35]	★★★☆	★☆	★☆☆	Fair
[36]	★★★☆	★☆	★★★	High
[18]	★☆☆☆	★★	★★☆	Fair
[37]	★★☆☆	★☆	★★☆	Fair
[19]	★★★☆	★★	★★☆	High
[44]	★☆☆☆	★☆	★★☆	Fair
[38]	★★★★	★☆	★★☆	High
[45]	★★★☆	★☆	★★☆	Fair
[20]	★★★☆	★★	★★☆	High
[]21	★☆☆☆	★☆	★★☆	Fair
[22]	★★★★	★☆	★★☆	High
[40]	★★★★	★☆	★★☆	High
[30]	★★★☆	★★	★★☆	High
[23]	★★★☆	★★	★★☆	High
[24]	★★★☆	★☆	★★☆	Fair
[31]	★★★☆	★☆	★★☆	Fair
[25]	★★☆☆	★★	★★☆	Fair
[32]	★★★☆	★☆	★★☆	Fair
[33]	★★★☆	★☆	★★☆	Fair

NOS: Newcastle-Ottawa scale;

★☆ Represents the number of points given for each domain to do the risk of bias scoring;

★ one point; ☆ zero point.
